# Unveiling Intestinal Emphysema in Pigs: Morphological Insights and Pathogenetic Implications

**DOI:** 10.3390/vetsci13010101

**Published:** 2026-01-20

**Authors:** Alfonso Rosamilia, Simona Baghini, Chiara Guarnieri, Anastasia Romano, Umberto Tosi, Giuseppe Marruchella, Attilio Corradi

**Affiliations:** 1 Istituto Zooprofilattico Sperimentale della Lombardia e dell’Emilia Romagna “Bruno Ubertini” (IZLER), 25124 Brescia, Italy; 2 Department of Veterinary Medicine, Località Piano d’Accio, University of Teramo, 64100 Teramo, Italyaromano@unite.it (A.R.);; 3 Local Health Unit Authority, 41121 Modena, Italy; 4 Department of Veterinary Science, University of Parma, Via del Taglio 10, 43126 Parma, Italy; attilio.corradi@unipr.it

**Keywords:** swine, slaughter, intestinal emphysema, microscopic features, lymphatic vessels

## Abstract

Intestinal emphysema is a rare pathological condition observed in humans and animals, characterized by the presence of multiple gas-filled cysts within the intestinal wall. In pigs, it is occasionally observed at slaughter, without affecting carcass suitability for human consumption or impairing farm profitability. Despite investigations, the etiology and pathogenesis of intestinal emphysema remain poorly understood. Therefore, this study aimed to provide further morphological insights into porcine intestinal emphysema through histopathological, histochemical and immunohistochemical methods. A total of ten slaughtered heavy pigs were examined, showing gross lesions consistent with intestinal emphysema. Gaseous cysts were predominantly located in the submucosal and mesenteric layers, at least partially lined by lymphatic endothelial cells. The cysts were separated by fibrous connective septa and were almost invariably associated with granulomas, consisting of epithelioid macrophages and multinucleated giant cells. Overall, the immunohistochemical patterns of porcine intestinal emphysema overlap with those described in humans and support the hypothesis that lesions likely originate within the lymphatic vessels.

## Simple Summary

Intestinal emphysema is characterized by gas-filled cysts within the intestinal wall, and it is occasionally observed in slaughtered pigs. The etiology and pathogenesis of intestinal emphysema remain poorly understood. This study aimed to provide further morphological insights into porcine intestinal emphysema through histopathological, histochemical and immunohistochemical methods. Ten slaughtered heavy pigs were examined, showing gross lesions consistent with intestinal emphysema. Gaseous cysts were mainly located in the submucosal and mesenteric layers, at least partially lined by lymphatic endothelial cells, and almost invariably associated with granulomas. Overall, data suggests that porcine intestinal emphysema is a lymphatic-centered disorder of the intestinal wall and mesentery, showing pathological features very similar to those described in human medicine.

## 1. Introduction

Intestinal emphysema (IE) is a disease condition observed in humans and animals, characterized by clusters of gas-filled cysts within the intestinal wall. Over time, this entity has been described using various terms—e.g., *pneumatosis cystoides intestinalis*, mesenteric emphysema, intestinal pneumatosis, and cystic lymphopneumatosis—reflecting its distinctive morphological features [1].

Intestinal emphysema primarily affects the small intestine and typically arises along the mesenteric border. However, IE may extend transmurally, involve the large intestine, and reach the mesenteric lymph nodes. Microscopically, IE lesions consist of variably sized, empty cystic spaces, which are located predominantly in the submucosa and mesentery, often surrounded by epithelioid macrophages and multinucleated giant cells [2,3,4].

In humans, IE is diagnosed incidentally in asymptomatic individuals or in patients presenting with nonspecific gastrointestinal signs [2,5,6,7,8]. In pigs, IE is occasionally detected at slaughter and appears to have no impact on public health, food safety, carcass suitability for human consumption, or farm profitability [4,9]. A recent survey reported prevalence values ranging from 1.25% to 5.12% in different slaughter batches, suggesting that porcine IE is more common than generally assumed [10]. Even much higher prevalence values have been rarely described under unusual environmental conditions [9].

Despite investigations, the etiology and pathogenesis of IE remain unclear. Several non-mutually exclusive hypotheses have been proposed, which may operate synergistically:

(a) Pulmonary theory—Chronic obstructive pulmonary disease (COPD) increases alveolar gas pressure, leading to rupture of the alveolar walls and septa, with air dissecting through the pulmonary interstitium into the mediastinum, the retroperitoneum, and ultimately the mesentery and the intestinal wall [8,11].
(b) Intestinal–mechanical theory—Increased intraluminal pressure and/or mucosal injury may allow gas to penetrate the intestinal wall, as observed in intestinal obstruction, hyperperistalsis, trauma, endoscopic insufflation, or surgical manipulation. Disruption of mucosal integrity may facilitate the passage of luminal gas into the submucosa or mesentery [5,12,13].
(c) Bacterial theory—Bacterial pathogens may contribute by damaging the intestinal mucosa and/or producing gas that accumulates within the intestinal wall. Experimental reproduction of IE in rats infected with *Clostridium perfringens* and in gnotobiotic pigs infected with *Escherichia coli* supports this hypothesis [14,15,16].
(d) Chemical–pharmacological theory—Administration of α-glucosidase inhibitors, immunosuppressive or chemotherapeutic agents, as well as occupational exposure to trichloroethylene, may enhance intraluminal gas production or alter mucosal permeability, thereby contributing to the development of IE [17].

This study aims to provide additional insights into the microscopic features of porcine IE, thus hopefully contributing to understand IE pathogenesis.

## 2. Materials and Methods

**Figure 1:**
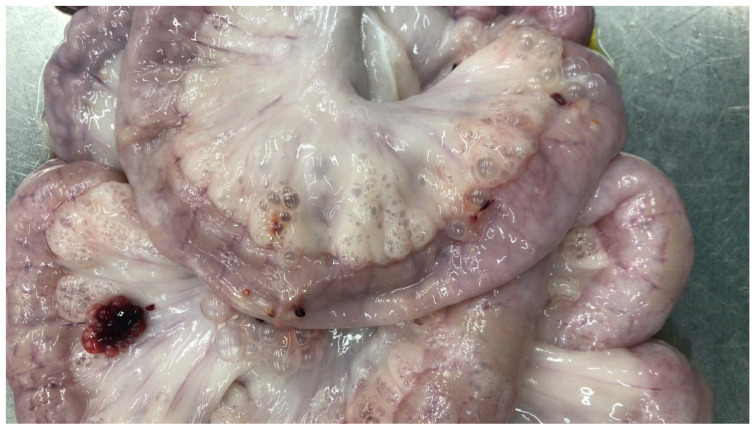
Pig. Ileum. Bubble-like appearance of the intestine, particularly along the mesenteric insertion and throughout the adjacent mesentery.

Ten heavy pigs (approximate slaughter weight of 160 kg, average age of 9–10 months), slaughtered at a commercial abattoir in Northern Italy and exhibiting gross IE lesions were included in this study (Figure 1). These pigs had been enrolled in a previous investigation [10], showed no relevant clinical sign at the *antemortem* inspection, and were carefully evaluated at *postmortem* inspection being admitted for human consumption in accordance with Regulation (UE) 2019/627. In particular, the gastrointestinal tract and pulmonary parenchyma showed no gross evidence of inflammatory lesions.

From each pig, a full-thickness, 2-cm-wide segment of the terminal ileum was collected, including the mesenteric border and the adjacent mesentery. Samples were immediately fixed in 10% neutral-buffered formalin, embedded in paraffin and routinely processed for histopathological evaluation (hematoxylin and eosin staining, H&E). An additional tissue section was subjected to *Masson*’s trichrome staining (Bio-Optica, Milan, Italy) to visualize fibrous connective tissue.

Given that biomolecular analyses performed on the same samples yielded inconclusive results and did not identify bacterial agents associated with granulomatous inflammation or necrotizing enteritis [10], additional histochemical investigations (e.g., Ziehl–Neelsen and Warthin–Starry staining) were not considered essential within the scope of this study.

Immunohistochemical analyses were performed to identify the cell types lining the cyst surface: lymphatic endothelial cells, macrophages, epithelial cells, and mesenchymal cells (see Table 1 for details). For this purpose, 4-µm-thick sections were mounted on positively charged slices (Bio-Optica, Milan, Italy), deparaffinized in xylene, and rehydrated through a graded ethanol series to distilled water. Heat-induced epitope retrieval was carried out in 0.01 M citrate buffer (pH 6.0) using a microwave oven at 750 W for 3 cycles of 5 min. Tissue sections were then cooled to room temperature and incubated for 20 min with a blocking solution (BLOXALL, Vector Laboratories, Newark, CA, USA) to inhibit endogenous peroxidase activity. Subsequently, the sections were incubated for 30 min with 2.5% normal horse serum (Vector Laboratories, Newark, CA, USA), followed by overnight incubation at 4 °C with the primary antibodies at their optimized working dilutions (Table 1). Immunoreactivity was visualized using a commercially available kit (ImmPRESS^®^ Polymer Detection Kit; Vector Laboratories, Newark, CA, USA) according to the manufacturer’s instructions, and the sections were finally counterstained with Mayer’s hematoxylin (Bio-Optica, Milan, Italy). Negative controls were included in each immunohistochemical run by omitting the primary antibody.

Histological and immunohistochemical slides were examined under a light microscope (Nikon Eclipse E800, Tokyo, Japan) at magnifications ranging from ×40 to ×400. Micrographs were captured using the DXM1200 digital camera (Nikon, Tokyo, Japan).

## 3. Results

**Figure 2:**
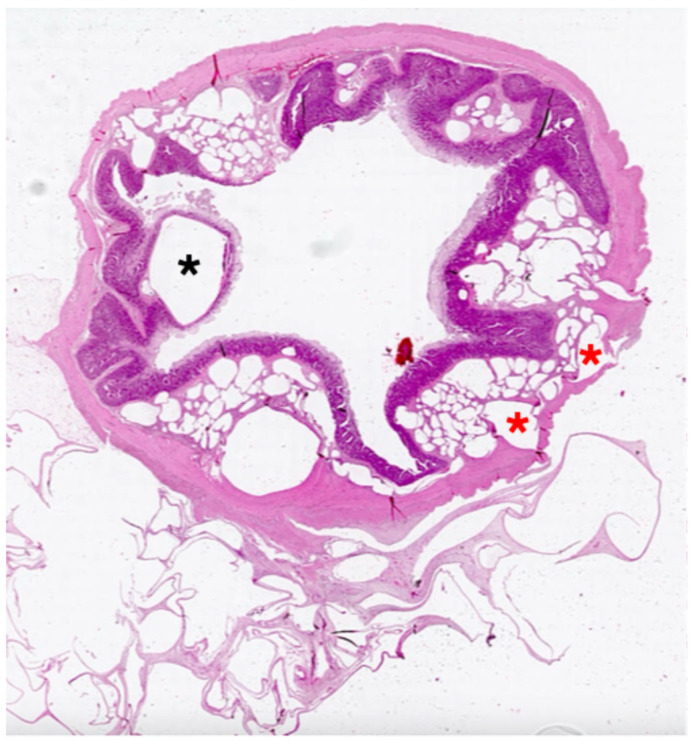
Pig. Ileum. A huge number of gas-filled cysts are seen within the mucosa (black asterisk), the submucosa, the muscular layer (red asterisks) and the mesentery. Hematoxylin and eosin stain. Final magnification ×10.

**Figure 3:**
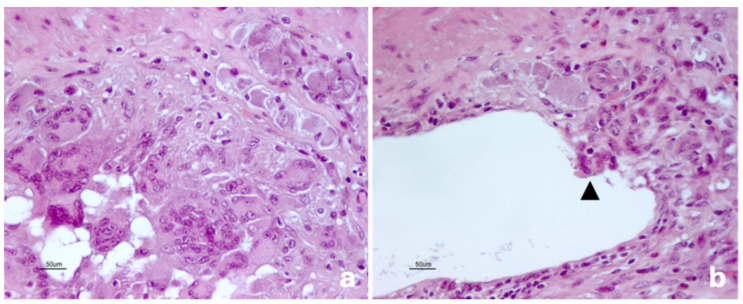
Pig. Ileum. Multinucleated giant cell-rich granuloma within the submucosal layer, close to an enteric nervous plexus (**a**). The inner surface of a cyst is lined by various cell types, including a multinucleated giant cell (black arrowhead; (**b**)). Hematoxylin and eosin stain.

A few cysts (2–15 *per* section) were observed within the mucosal layer of three pigs. Submucosal involvement was consistent and characterized by numerous cysts (>50 per section), some of which were large and caused atrophy of the adjacent tissues (Figure 2). The cyst luminal surface was lined by heterogeneous cellular populations—i.e., endothelium-like cells, epithelioid cells, and multinucleated giant cells—which frequently co-occurred within the same lesion (Figure 3a,b). Granulomas and lymphocytic aggregates were common within the submucosa and inside the connective septa separating the cystic structures.

Gas-filled cysts were less frequently observed within the tunica muscularis. The mesentery represented the most severely affected anatomical district, containing an extremely high, often countless burden of cysts. Microscopic features of cysts resembled those observed within the submucosa.

**Figure 4:**
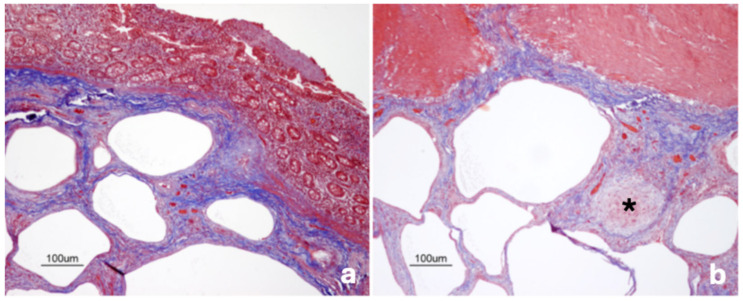
Pig. Ileum. Septa delimitating cysts are rich in collagen fibers (blue stained; (**a**)) and contain giant cell-rich granuloma (black asterisk; (**b**)). *Masson*’s trichrome stain.

*Masson*’s trichrome staining showed that septa delineating the cysts—particularly those in the submucosa and mesentery—mainly consisted of dense collagenous stroma. In these same layers (i.e., submucosa and mesentery), a prominent fibrotic reaction also surrounded granulomas enriched in multinucleated giant cells (Figure 4a,b).

**Figure 5:**
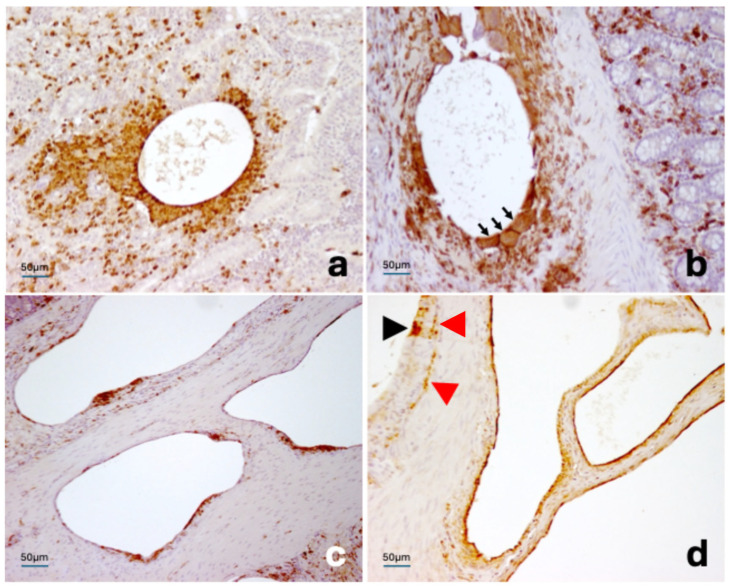
Pig. Ileum. Immunohistochemistry. A mucosal cyst is surrounded by a dense infiltrate of macrophages immunoreactive for MAC387 (**a**). Several IBA1/AIF-positive multinucleated giant cells (black arrows) are present around a cyst within the submucosa (**b**). IBA1/AIF-positive cells also partially line the inner surface of cysts located in the muscular layer (**c**). LYVE-1-immunoreactive cells line the mesenteric cysts; a single LYVE-1-positive epithelioid cell is additionally observed contributing to the lining of one cyst (black arrowhead). A thin and linear immunolabelling for LYVE-1 is seen within the pericystic interstitium (red arrowheads), which is separated from the cystic lumen by multiple layers of LYVE-1-negative cells (**d**). Mayer’s hematoxylin counterstain.

Immunohistochemical investigations pointed out that the cysts were variably lined by lymphatic endothelial cells (LYVE-1–immunoreactive), macrophages (immunoreactive for vimentin, MAC387, and IBA1/AIF1), and multinucleated giant cells (immunoreactive for vimentin and IBA1/AIF1, with inconsistent or weak MAC387-immunolabeling). Aggregates of MAC387-positive cells were observed more frequently within the lamina propria, where they tended to cluster around the cysts. LYVE-1-positive macrophages cells were also noted on occasion. In addition, a layer of epithelioid cells was sometimes present between the lymphatic endothelium and the cystic lumen (Figure 5a–d). Intestinal epithelial cells and mesothelial cells showed consistent and intense immunolabeling for cytokeratin. Mesothelial cells were infrequently observed in direct apposition to the surfaces of gas-filled cysts, apparently contributing to the architecture of the cyst wall.

## 4. Discussion

This study does not resolve the enigmatic nature of IE, as its etiology and pathogenesis remain largely obscure. In addition, some inherent limitations should be acknowledged, primarily related to the limited sample size and the features of the samples, which likely reflect the end stage of a long, complex, and multifactorial process. Nonetheless, it provides a detailed morphological description of porcine IE, allowing some plausible considerations.

Histopathological and immunohistochemical findings support the hypothesis that gas-filled cysts originate from lymphatic vessels. This interpretation is consistent with the topographical distribution of lesions, which mainly affect the submucosa and mesentery, anatomical compartments rich in lymphatic vessels. Moreover, it is further corroborated by the identification of LYVE-1-positive endothelial cells lining the inner surface of the cysts. These findings align closely with those reported in humans by Gui et al. [1], who concluded that “*the lymphatic theory is most likely valid… and the lymphatic vessels represent the common pathway for gas transport in various conditions in which the integrity of the intestinal mucosa is compromised*”.

Overall, the immunohistochemical patterns of porcine IE mirrored those described in humans [1], even though different markers were employed. Lymphatic endothelial cells inconsistently and/or partially lined the cystic lumina and were frequently replaced by MAC387 and/or IBA1/AIF1-positive cells. Epithelioid macrophages and multinucleated giant cells were often interposed between LYVE-1-positive endothelial cells and the cystic cavity, and occasionally exhibited LYVE-1 immunoreactivity themselves. In this respect, Gui et al. [1], hypothesized that gaseous dilation may damage lymphatic endothelial cells, thereby triggering a chronic inflammatory response. Macrophages may subsequently accumulate around the lymphatics and phagocytose injured endothelial cells, becoming transiently immunoreactive for lymphatic markers (e.g., LYVE-1). We consider this mechanism plausible in humans and reasonably applicable to porcine IE as well.

In our opinion, the “pulmonary theory”—primarily formulated in humans—is unlikely to account for IE in pigs, as the pathogenesis of porcine chronic bronchopneumonia (e.g., caused by *Mycoplasma hyopneumoniae*) is entirely distinct from that of human COPD, and it does not involve marked emphysematous change [8,11,20]. Similarly, other hypotheses—e.g., post-surgery occurrence of IE or chemical–pharmacological theory—are conceivable in humans or companion animals [12,13,17], but they cannot reasonably be extended to reared pigs.

*Escherichia coli* has been shown to induce IE in gnotobiotic pigs [14], and this finding supports the “bacterial theory” as a plausible explanation for porcine IE. Nevertheless, in-depth analyses of the gut microbiota have failed to identify a consistent bacterial pathogen or dysbiosis signature in IE-affected pigs. Speculatively, non-mutually exclusive explanations may account for this observation. For instance, a bacterial pathogen may act in a “hit-and-run” manner, being no longer detectable at the time of slaughter [10]. Alternatively, the mucosal integrity may be compromised by multiple pathogens and/or by additional concurrent factors. In this context, we have recently experienced cases of colonic IE in growing pigs with swine dysentery; these lesions likely result from *Brachyspira hyodysenteriae*-induced necrosis, they lack granulomatous reactions and may represent an early stage of IE (unpublished data). The potential association between IE and specific intestinal diseases, particularly those characterized by necrotizing lesions (e.g., swine dysentery, proliferative ileitis, salmonellosis), warrants further investigation. If substantiated, IE could be regarded as a sentinel lesion, contributing to offer valuable feedback for herd health monitoring.

Notably, cysts are often surrounded by MAC387-immunoreactive, indicative of recent recruitment [21]. This finding is particularly evident within the mucosal layer, suggesting that IE is a long-standing inflammatory and fibroproliferative process, which continues to progress over time and possibly originates at the mucosal interface.

In conclusion, this study supports the view that porcine IE is a chronic, lymphatic-centered disorder. Considering its pathological features and according to the literature [1], the following scenario may be envisaged: (1) a transient, yet unidentified, insult perturbs the lymphatic function or integrity within the gut wall; (2) impaired lymphatic drainage or structural damage of lymphatic vessels predisposes to the accumulation of gas; (3) gaseous distension further damages the lymphatic endothelium, triggering macrophages recruitment and granuloma formation; (4) persistent, low-grade inflammation leads to fibrosis, partial replacement of the lymphatic endothelium by macrophages, and ultimately the formation of stable gas-filled cysts. The parallels between porcine and human IE suggest shared pathogenetic mechanisms across species and underscore the value of pigs as a comparative model. Deeper and prospective investigations of potential risk factors (e.g., dietary components, microbiota composition, genetic background) are warranted for elucidating the etiology and pathogenesis of IE. Finally, additional insights may be gained from the analysis of the gas composition within the cysts, an approach that is already planned for future investigations. Comparative evaluation of gas composition data with those available from human pathology [22] may contribute to a better understanding of the pathogenesis of porcine IE and further clarify the extent to which it mirrors the human condition.

## Figures and Tables

**Table 1:** Immunohistochemical analyses. Technical details about cell types, antigenic targets, and primary antibodies.

Antigen	Target Cells	Primary Antibody	Working Dilution
LYVE-1	Endothelial cells of lymphatic vessels	Rabbit polyclonal antibody. The exact immunogen used to generate this antibody is not available (Abcam, Cambridge, UK; ab33682) ^a^	1:200
MAC387	Macrophages	Mouse monoclonal antibody raised against human peripheral blood monocyte components (Santa Cruz Biotechnology, Dallas, TX, USA; sc-66204) ^b^	1:50
IBA1/AIF1	Macrophages	Mouse monoclonal antibody raised against human allograft inflammatory factor 1 isoform 3 (Millipore, Burlington, MA, USA; MABN92) ^c^	1:400
Cytokeratins	Epithelial cells	Rabbit polyclonal antibody raised against native full length protein corresponding to cow KRT5 (Abcam, Cambridge, UK; ab9377) ^d^	1:100
Vimentin	Mesenchymal cells	Rabbit polyclonal antibody. The exact immunogen used to generate this antibody is not available. The manufacturer predicts reactivity in pigs based on strong sequence homology (Abcam, Cambridge, UK; ab45939) ^e^	1:100

^a^ This primary antibody has previously been used in pigs for immunohistochemistry [18]. Lacteals within the intestinal villi served as internal positive controls. ^b^ This primary antibody has been preliminary assessed in tissue sections of porcine lung and lymph node, which also served as positive controls. ^c^ This primary antibody has previously been validated in pigs by Western blot analysis [19]. It has been preliminary assessed in tissue sections of porcine lung and lymph node, which also served as positive controls. ^d^ According to the manufacturer, this primary antibody has been tested in pigs. Enterocytes served as internal positive controls. ^e^ Mesenchymal cells within the intestinal wall and porcine skin sections served as positive controls.

## Data Availability

The original contributions presented in the study are included in the article. Further inquiries can be directed to the corresponding author.

## Abbreviations

The following abbreviations are used in this manuscript:

IE	Intestinal emphysema
COPD	Chronic obstructive pulmonary disease
H&E	Hematoxylin and eosin
